# Effective Use of Olanzapine for Chemotherapy-Induced Nausea and Vomiting in a Patient Receiving Dose-Adjusted Rituximab, Etoposide, Prednisone, Vincristine (Oncovin®), Cyclophosphamide, and Doxorubicin (R-EPOCH): A Case Report

**DOI:** 10.7759/cureus.99657

**Published:** 2025-12-19

**Authors:** Hirokazu Shimada, Masahiro Ohgami, Yasumitsu Kurokawa, Shigeki Tachihara, Akinori Sugaya

**Affiliations:** 1 Department of Pharmacy, Ibaraki Prefectural Central Hospital, Kasama, JPN; 2 Department of Hematology, Ibaraki Prefectural Central Hospital, Kasama, JPN; 3 Department of Medical Oncology, Ibaraki Prefectural Central Hospital, Kasama, JPN

**Keywords:** antiemetics, chemotherapy-induced nausea and vomiting (cinv), da-epoch-r, olanzapine, supportive care

## Abstract

Dose-adjusted (DA) rituximab, etoposide, prednisone, vincristine (Oncovin^®^), cyclophosphamide, and doxorubicin (R-EPOCH) is a multiday chemotherapy regimen that includes anthracyclines and cyclophosphamide. Although olanzapine is used to prevent chemotherapy-induced nausea and vomiting (CINV), its efficacy in patients receiving DA-R-EPOCH has not yet been reported. We describe a case in which olanzapine was effective in preventing CINV during DA-R-EPOCH treatment. A 21-year-old woman was diagnosed with primary mediastinal large B-cell lymphoma. The patient had some CINV risk factors associated with a lower risk, such as no history of motion sickness or morning sickness (no history of pregnancy), as well as factors associated with a higher CINV risk, including a lack of habitual alcohol consumption and no history of smoking. The patient received prophylactic antiemetic therapy with olanzapine (5 mg after dinner for six days), palonosetron, and fosnetupitant during DA-R-EPOCH therapy. Prednisolone was administered at 60 mg/m^2^ for five days as part of the DA-R-EPOCH regimen; it also served as a corticosteroid with antiemetic activity. From days 1 to 10 of treatment, the food intake rate remained above 70%. Grade 1 nausea was observed on days 4 and 5, with no vomiting during the treatment period. Adverse events, such as constipation and somnolence, were also noted. A four-drug antiemetic regimen, including olanzapine, supported the feasibility of preventing CINV in this case involving DA-R-EPOCH therapy. However, adverse events potentially related to olanzapine were observed, suggesting the need for further investigation into its optimal dosage.

## Introduction

Nausea and vomiting are among the most common adverse events associated with chemotherapy and can significantly impair the quality of life (QOL) of patients with cancer [1]. Chemotherapy-induced nausea and vomiting (CINV) is graded according to the Common Terminology Criteria for Adverse Events (CTCAE), with grade 1 defined as mild (loss of appetite without alteration in eating habits), grade 2 as moderate (decreased oral intake without significant weight loss), and grade 3 as severe (inadequate oral caloric or fluid intake; tube feeding or hospitalization indicated) [2]. Moreover, grade 3 or higher severe CINV is associated with a worse prognosis, highlighting the importance of effective prophylactic antiemetic therapy [3]. CINV is categorized into four levels based on the emetogenic potential of chemotherapeutic agents without prophylactic treatment. Regimens that combine anthracyclines and cyclophosphamide are considered highly emetogenic (>90% risk of vomiting). Recently, olanzapine has been shown to be effective in preventing CINV [4]. It exerts an antiemetic effect by antagonizing multiple receptors, including dopamine, serotonin, adrenergic, muscarinic, and histaminic receptors [5]. Accordingly, clinical guidelines, such as those from the American Society of Clinical Oncology (ASCO) and the Multinational Association of Supportive Care in Cancer (MASCC)/European Society of Medical Oncology (ESMO), recommend a four-drug antiemetic regimen comprising olanzapine, a 5-hydroxytryptamine-3 (5-HT_3_) receptor antagonist, a neurokinin-1 (NK_1_) receptor antagonist, and a corticosteroid for highly emetogenic chemotherapy [6,7]. However, evidence supporting this regimen for anthracycline and cyclophosphamide-containing protocols primarily comes from studies in patients with breast cancer [8], with limited data on other malignancies.

Dose-adjusted (DA) rituximab, etoposide, prednisone, vincristine (Oncovin^®^), cyclophosphamide, and doxorubicin (R-EPOCH) is a multiday chemotherapy regimen containing anthracycline and cyclophosphamide, developed to enhance therapeutic efficacy through dose escalation based on hematologic tolerance [9]. Effective management of adverse events, including CINV, is essential for enabling such escalation. Previous studies have shown that a combination of a 5-HT_3_ receptor antagonist and corticosteroid alone is insufficient to prevent CINV during DA-R-EPOCH [10,11]. Although the addition of an NK_1_ receptor antagonist improved the complete response (CR) rate, defined as no vomiting and no use of rescue medication, the CR rate remained at 57.1% [12]. Although the ASCO and MASCC/ESMO guidelines recommend a four-drug antiemetic regimen for highly emetogenic chemotherapy [6,7], no studies have specifically evaluated the efficacy of olanzapine in DA-R-EPOCH. These guidelines recommend administering olanzapine for four days in regimens in which chemotherapy is administered on day 1 only. However, DA-R-EPOCH therapy is a multiday regimen in which doxorubicin is administered on days 2-5 and cyclophosphamide on day 6 [9], raising the possibility that nausea and vomiting may persist longer than that in single-day anthracycline/cyclophosphamide regimens used for breast cancer [8]. In addition, previous studies of multiday cisplatin-based regimens have reported extending olanzapine administration beyond the guideline-recommended four days [13]. Herein, we report a case in which a four-drug antiemetic regimen, including olanzapine administration for six consecutive days to cover this prolonged risk period, prevented CINV during DA-R-EPOCH therapy.

## Case presentation

A 21-year-old woman was admitted to initiate DA-R-EPOCH therapy following a diagnosis of primary mediastinal large B-cell lymphoma. The patient had no relevant medical history or comorbidities and was not taking any prescription drugs, over-the-counter medications, or supplements before admission. To prevent tumor lysis syndrome and peptic ulcers, febuxostat (20 mg once daily after breakfast) and esomeprazole (10 mg once daily after breakfast) were initiated. The patient had some CINV risk factors associated with a lower risk, such as no history of motion sickness or morning sickness (no history of pregnancy), as well as factors associated with a higher CINV risk, including a lack of habitual alcohol consumption and no history of smoking. The serum calcium and sodium levels were within normal limits. The administration schedule for DA-R-EPOCH and antiemetic agents is shown in Table 1. DA-R-EPOCH was initiated at level 1 in accordance with the protocols of previous clinical trials for primary mediastinal large B-cell lymphoma [14]. The prophylactic antiemetics included oral olanzapine (5 mg once daily after dinner on days 2-7), intravenous palonosetron (0.75 mg on day 2), and intravenous fosnetupitant (235 mg on day 2). Intravenous prednisolone (60 mg/m^2^ once daily on days 2-6) was administered as part of the DA-R-EPOCH regimen; it also functioned as a corticosteroid with antiemetic activity.

**Table 1:** Dosing schedule of dose-adjusted R-EPOCH (level 1) and antiemetic therapy * If the nadir absolute neutrophil count is ≥500/µL, dose levels of etoposide, doxorubicin, and cyclophosphamide were increased by 20% from the previous cycle. Arrows (↑) indicate the timing of drug administration. R-EPOCH: rituximab, etoposide, prednisone, vincristine (Oncovin^®^), cyclophosphamide, and doxorubicin

Medications	Dose	Days						
		1	2	3	4	5	6	7
DA-R-EPOCH (level 1)	-	-	-	-	-	-	-	-
Rituximab	375 mg/m^2^	↑	-	-	-	-	-	-
Etoposide	50 mg/m^2^ *	-	↑	↑	↑	↑	-	-
Vincristine	0.4 mg/m^2^	-	↑	↑	↑	↑	-	-
Doxorubicin	10 mg/m^2^ *	-	↑	↑	↑	↑	-	-
Cyclophosphamide	750 mg/m^2 ^*	-	-	-	-	-	↑	-
Prednisolone	60 mg/m^2^	-	↑	↑	↑	↑	↑	-
Antiemetics	-	-	-	-	-	-	-	-
Olanzapine	5 mg	-	↑	↑	↑	↑	↑	↑
Palonosetron	0.75 mg	-	↑	-	-	-	-	-
Fosnetupitant	235 mg	-	↑	-	-	-	-	-

CINV and other adverse events are shown in Table 2. The food intake rate was defined as the percentage of average daily intake for each meal, based on estimates recorded by the nurse at the time of dish collection. Her food intake rate remained above 70% from day 1 to 10. Grade 1 nausea occurred on days 4 and 5, based on the CTCAE version 5.0 [2], and a single rescue dose of metoclopramide (5 mg) was administered on day 4. There were no vomiting episodes during treatment; furthermore, no clinically significant weight loss was observed. The patient experienced reduced bowel movements on days 4 and 5; therefore, sennoside (12 mg once daily at bedtime, days 5-6) and lubiprostone (24 μg twice daily after meals, days 5-8) were administered. Somnolence was assessed by interviewing the patient about the degree of daytime sleepiness and its effect on daily life, and graded it according to the CTCAE criteria: grade 2 on day 3 and grade 1 on days 4-8. Additionally, fatigue (grade 2 on day 6) and dysgeusia (grade 1 on day 4) were observed. As CINV was well controlled, the DA-R-EPOCH dose was successfully increased to level 3 in accordance with the protocol, supported by nadir absolute neutrophil counts of 2,400/μL during the first cycle and 2,300/μL during the second cycle.

**Table 2:** Adverse events after dose-adjusted R-EPOCH treatment The food intake rate was defined as the percentage of average daily intake for each meal, based on estimates recorded by the nurse at the time of dish collection. Adverse events were graded according to the Common Terminology Criteria for Adverse Events (CTCAE) version 5.0. CINV: chemotherapy-induced nausea and vomiting; R-EPOCH: rituximab, etoposide, prednisone, vincristine (Oncovin^®^), cyclophosphamide, and doxorubicin; arrows (↑) indicate the timing of drug administration; circles (〇) indicate the days when adverse effects occurred.

Adverse events		Days									
		1	2	3	4	5	6	7	8	9	10
CINV		-	-	-	-	-	-	-	-	-	-
Food intake rate (%)		92	100	92	75	83	90	95	88	77	97
Nausea	Grade 1	-	-	-	○	○	-	-	-	-	-
Rescue dose (metoclopramide (5 mg))		-	-	-	↑	-	-	-	-	-	-
Number of vomiting episodes		0	0	0	0	0	0	0	0	0	0
Body weight (kg)		65.5	65.9	66.6	67.4	66.8	66.4	65.8	65.3	64.5	64.6
Constipation		-	-	-	-	-	-	-	-	-	-
Number of bowel movements		1	1	1	0	0	1	2	1	2	3
Sennoside (12 mg once daily)		-	-	-	-	↑	↑	-	-	-	-
Lubiprostone (24 μg twice daily)		-	-	-	-	↑	↑	↑	↑	-	-
The others		-	-	-	-	-	-	-	-	-	-
Somnolence	Grade 2	-	-	○	-	-	-	-	-	-	-
	Grade 1	-	-	-	○	○	○	○	○	-	-
Fatigue	Grade 2	-	-	-	-	-	○	-	-	-	-
Dysgeusia	Grade 1	-	-	-	○	-	-	-	-	-	-

## Discussion

This case highlights two key findings. First, the combination of olanzapine, a 5-HT_3_ receptor antagonist, an NK_1_ receptor antagonist, and a corticosteroid was effective in preventing CINV during DA-R-EPOCH. Second, appropriate management of non-CINV adverse events is essential during this regimen.

The patient maintained a food intake rate of over 70%, experienced only grade 1 nausea, and did not vomit (Table 2). Previous studies have shown that four-drug antiemetic regimens, including olanzapine, are effective in preventing CINV during multiday chemotherapy for hematopoietic cell transplantation [15]. In this case, the same approach appeared effective during DA-R-EPOCH, another multiday chemotherapy regimen involving anthracyclines and cyclophosphamide. In DA-R-EPOCH therapy, prednisolone is administered at 60 mg/m^2^ daily for five days (Table 1), which is approximately equivalent to 9 mg/m^2^ dexamethasone [16]. For highly emetogenic regimens in which chemotherapy is administered only on day 1, the ASCO and MASCC/ESMO guidelines recommend dexamethasone at 12 mg on day 1 and 8 mg on days 2-4 [6,7]. Therefore, the cumulative steroid dose in DA-R-EPOCH therapy is considerably higher than that recommended in current guidelines, which may have contributed to the good control of CINV in this case. However, previous studies of DA-R-EPOCH have shown that a 5-HT_3_ receptor antagonist, corticosteroid, and NK_1_ receptor antagonist alone often fail to adequately prevent CINV, with CR rates remaining insufficient [12]. Therefore, olanzapine may have contributed to the adequate control of CINV in our patient.

It is also essential to manage non-CINV adverse events appropriately during this regimen. In the present case, the patient developed constipation on days 4 and 5 (Table 2). Vincristine, a microtubule inhibitor included in DA-R-EPOCH, is known to cause constipation, with a reported incidence of 4% [9]. Olanzapine and palonosetron also contribute to constipation [17,18]. Laxatives effectively restored bowel movements by day 6, and food intake subsequently improved. Although a causal relationship cannot be confirmed, appropriate management of constipation may have contributed to the alleviation of CINV symptoms. Somnolence was observed as grade 2 on day 3 and grade 1 on days 4-8, likely associated with olanzapine (Table 2). Olanzapine is an antipsychotic agent that blocks various receptors, with somnolence mainly caused by its strong antagonism of the histamine receptors [5]. Based on a previous report, olanzapine was administered after dinner in this case so that its plasma concentration would peak during the patient’s usual sleep period [17], but grade 2 somnolence still occurred. Recent trials have reported that 2.5 mg olanzapine may also be effective in preventing CINV while minimizing somnolence [19]. Although a reduced dose of olanzapine is often recommended for older adults at high risk for daytime somnolence, vincristine, a component of DA-R-EPOCH therapy, can cause both acute and chronic neurotoxicity that interferes with activities of daily living, regardless of age [20]. Therefore, even in younger patients, using a lower dose of olanzapine (e.g., 2.5 mg) may be a reasonable option to avoid exacerbating functional impairment during therapy.

In this case, palonosetron was used as a 5-HT_3_ receptor antagonist, and fosnetupitant was used as an NK_1_ receptor antagonist (Table 1). Palonosetron, a second-generation 5-HT_3_ receptor antagonist, has a uniquely strong binding affinity for the receptor and has been shown to more effectively reduce delayed-phase CINV compared to first-generation 5-HT_3_ receptor antagonists [18]. Fosnetupitant is converted to its active metabolite, netupitant, which has a high affinity and selectivity for NK_1_ receptors [21]. In regimens including cisplatin or anthracycline-cyclophosphamide combinations, fosnetupitant has shown antiemetic efficacy equivalent to that of fosaprepitant and may offer prolonged protection beyond 120 hours after chemotherapy initiation [22,23]. Given the extended CINV risk associated with multiday chemotherapy, such as DA-R-EPOCH, the combination of palonosetron and fosnetupitant may represent a particularly effective antiemetic strategy.

## Conclusions

This case suggests that a four-drug antiemetic regimen, including olanzapine, a 5-HT_3_ receptor antagonist, an NK_1_ receptor antagonist, and a corticosteroid, supported the feasibility of preventing CINV during DA-R-EPOCH therapy. Additionally, managing non-CINV adverse effects, such as constipation and somnolence, may have helped maintain food intake and contributed to improving overall patient outcomes. CINV is a common chemotherapy side effect that impacts QOL and interferes with dose escalation according to the DA-R-EPOCH protocol, making appropriate antiemetic prophylaxis crucial for patient well-being and the successful delivery of treatment. Further studies are warranted to determine the optimal dosage and duration of olanzapine for balancing effectiveness and tolerability in multiday chemotherapy regimens, including prospective evaluations in patients receiving DA-R-EPOCH therapy that incorporate standardized tools of patient-reported outcomes, such as the Functional Living Index Emesis and the MASCC Antiemesis Tool questionnaires.
